# Effect of different splinting configurations on the accuracy of complete-arch digital implant impressions: An in vitro study

**DOI:** 10.4317/jced.64318

**Published:** 2026-08-28

**Authors:** Simge Öner-Gedik, Helin Yolcu, Pınar Türkoğlu, Ali Murat Kökat

**Affiliations:** 1 Department of Prosthodontics, Faculty of Dentistry, Istanbul Aydın University, Istanbul, Turkey

**Keywords:** Scanbody splinting, digital implant impression, trueness, precision, complete arch.

## Abstract

**Background:**

This in vitro study aimed to investigate the effect of different splinting configurations on the accuracy of digital impressions in fully edentulous arches.

**Materials and Methods:**

Five configurations were tested on an edentulous maxillary phantom model containing six parallel implant analogs: G1: Non-splinted control, G2: Bilateral posterior splint, G3: Anterior splinted/posterior unsplinted, G4: Segmental independent splinting, and G5: Cross-arch. Ten scans were obtained per group; reference data were acquired using a high-accuracy industrial scanner (Solutionix C500). Point-based RMS (Root Mean Square), surface RMS, and angular deviation (ΔAngle) were calculated using Geomagic Control X. ART ANOVA and one-way ANOVA with Bonferroni-corrected post-hoc tests were applied (α=0.05).

**Results:**

Significant differences among groups were detected for all parameters (p<0.001). In surface RMS, G5 exhibited the lowest deviation (0.62 ± 0.15 mm) and G2 the highest (1.27 ± 0.10 mm) (ηp^2^=0.793). In point-based RMS, G4 showed significantly higher deviation compared to G2, G3, and G5 (0.05 vs. 0.02-0.04mm). Regarding angular deviation, G2 showed the highest and G5 the lowest values (0.26° vs. 0.16°); the anterior and premolar regions demonstrated significantly higher angular deviation than the molar region (0.29-0.34° vs. 0.14°).

**Conclusions:**

The effect of splinting on scanning accuracy was found to be parameter-dependent; G5 was superior in surface RMS and angular deviation, yet no configuration achieved consistent superiority across all parameters. The anterior-premolar region was identified as the highest-risk area for accuracy across all configurations, exhibiting the greatest angular deviation values.

## Introduction

Success in implant-supported prosthetic treatment depends on the accurate transfer of the three-dimensional positions of implants to the working cast and the achievement of a passively fitting superstructure [1]. Since osseointegrated implants lack the buffering effect of the periodontal ligament, impression errors can lead to mechanical and biological complications such as screw loosening, prosthesis fracture, and marginal bone loss [1]. Therefore, impression accuracy is one of the fundamental factors determining the long-term success of implant-supported restorations [1]. Although conventional impression techniques have long been accepted as the gold standard, dimensional changes in impression materials, casting procedures, and multi-step laboratory processes can cause cumulative errors; furthermore, the adverse effect of impression materials on patient comfort constitutes a significant disadvantage [2-4]. To address these problems, digital workflows have become widespread with the development of intraoral scanner (IOS) use. Digital impression methods are considered advantageous because they reduce the risk of dimensional distortion, eliminate laboratory-related error steps, and improve patient comfort [5-8]. However, the accuracy of IOS in fully edentulous arches remains a subject of debate; the long edentulous spans between implants and the movable mucosa lacking sufficient anatomical reference points increase cumulative deviations during image stitching, thereby reducing accuracy in full-arch scans [4,6,9-11]. For this reason, conventional impressions have been reported to yield more reliable results in full-arch cases [12].

According to ISO 5725, accuracy is composed of trueness and precision [13]. Various strategies have been proposed to improve these parameters in fully edentulous ridges, including the use of auxiliary geometric devices, photogrammetry-based hybrid methods, and splinting of scan bodies [2,14]. The splinting approach aims to reduce micro-movements by creating a rigid connection between scan bodies and to provide more stable reference points for the image stitching algorithm [12]. However, evidence in the literature regarding the effect of different splinting materials and connection protocols on accuracy remains insufficient [3,15]. The Straumann EXACT™ system (Institut Straumann AG, Basel, Switzerland) enables BASE scanbodies to be connected to one another via LINK components to achieve a more stable structure, thereby aiming to improve impression accuracy and passive fit without the need for additional equipment such as photogrammetry [16]. However, due to implant positions, arch geometry, and clinical conditions, it may not always be feasible to apply the standard LINK configuration uniformly in every case. While the Straumann EXACT™ system is increasingly used in clinical practice, the effect of different LINK connection sequences on intraoral scanning accuracy has not been previously investigated. Clinicians are often required to modify the standard connection protocol based on patient-specific implant positions, yet the accuracy implications of such variations remain unknown. The aim of this study was to compare the effect of different LINK connection sequences in an All-on-6 configuration on intraoral scanning accuracy. The null hypothesis of this study was that there would be no significant difference in scanning accuracy among the different splinting configurations.

## Materials and Methods

*Study Design*

This study was designed as an *in vitro* experimental investigation. Since no human or animal material was used, ethics committee approval and informed consent were not required. Sample size was determined using G*Power 3.1 (Heinrich Heine University, Düsseldorf, Germany) based on a comparable study [6,17]. For the one-way ANOVA model, the effect size Cohen's *F=*1.29, α=0.05, and power (1-β)=0.80 as reported in a similar study were used. Analysis yielded a minimum sample size of 15. To increase measurement reliability and control inter-group variability, 10 repetitions per group were planned, resulting in a total sample size of 50.

*Model Preparation*

The reference master model was prepared on an edentulous maxillary phantom model (Fuji Elite SE32JAW, Nissin Dental Products Inc., Kyoto, Japan). Teeth were removed to create a fully edentulous arch, and extraction sockets were filled with modeling wax to simulate soft tissue. Implant analogs (REF: 023.4756, Straumann Holding AG, Basel, Switzerland) were placed at positions 12, 14, 16, 22, 24, and 26. Open-tray impression posts were positioned using a parallelometer (DeguDent F4 Basic, Dentsply Sirona, Bensheim, Germany), and the entire assembly was rigidly stabilized with modeling acrylic (Pattern Resin LS, GC America, Alsip, IL, USA). An impression was taken using the open-tray technique and type A silicone impression material (Variotime Easy Putty, Kulzer GmbH, Hanau, Germany); following polymerization, analogs were seated into their corresponding positions within the impression. The cast was fabricated using type IV hard gypsum (SilaDent Marmorock E, SilaDent Dr. Böhme & Schöps GmbH, Germany) with a dental vibrator (Pulse-4, TD Dental, Istanbul, Turkey) in accordance with the manufacturer's instructions. The resulting model was used as the reference master model for all configurations.

*Acquisition of the Reference Dataset*

The reference dataset was obtained by placing only BASE scanbodies (REF: 025.0084, Straumann Holding AG, Basel, Switzerland) on the implant analogs in accordance with the manufacturer's instructions, without applying any splinting. This configuration was scanned once using a high-accuracy laboratory scanner (Solutionix C500, Medit Corp., Seoul, Republic of Korea), and the resulting STL file was accepted as the reference model. The device was validated according to VDI/VDE 2634 Part 2 and Part 3 standards, with a reported mean measurement error of 1 µm; therefore, a single scan was deemed sufficient for the reference dataset. The scanning volume was 136 × 111 × 60 mm, and calibration was performed prior to scanning in accordance with the manufacturer's instructions.

*Definition of Splinting Configurations*

Five groups were established in the study. In Groups 2-5, LINK components were applied in accordance with the manufacturer's instructions and secured with flowable composite resin (RubyFlow, Rubydent, Istanbul, Turkey). Polymerization was performed using an LED curing unit (3M™ Elipar™ DeepCure-S, 3M ESPE, St. Paul, MN, USA; 1470 mW/cm²) per the manufacturer's instructions. All splinting configurations used in the study are presented in Table 1 and Figure 1.

*Digital Scanning Protocol*

A single reference model was used throughout the study, with all configurations applied sequentially to this model. After each configuration, the flowable composite resin connecting the LINK components was removed, and the LINK components and BASE scanbodies were detached. BASE scanbodies were reattached using a Straumann torque wrench (Straumann Holding AG, Basel, Switzerland) in accordance with the manufacturer's instructions, after which the next configuration was applied. Digital scans were performed using an intraoral scanner (Straumann SIRIOS™, ST200E, Straumann Holding AG, Basel, Switzerland) and Straumann SIRIOS™ software (version 1.0.6). All scans were performed by a single experienced operator under constant lighting conditions and at 23 ± 1 °C, in separate sessions to minimize operator adaptation and fatigue effects. During scanning, the operator began from the left posterior region and continuously scanned the occlusal, then buccal and palatal surfaces. Prior to each scan, the seating of the BASE scanbodies was verified.

*Data Analysis and Superimposition*

STL datasets were imported into Geomagic Control X software (2021.3, 3D Systems, Rock Hill, SC, USA). After loading the reference model, five points were manually marked at the cervical region of each implant and used as fixed references in all alignments. The cervical region was selected as it provides a stable and reproducible reference surface, and these points were not altered in subsequent datasets. The alignment process was initiated with initial alignment and completed using the best-fit alignment algorithm. Point-based RMS deviations were automatically calculated by the software; additionally, cylindrical geometries were constructed for each implant to obtain angular deviations and surface RMS deviation values. To account for the systematic measurement error of the scanner, the mean deviation values (point-based RMS, surface RMS, ΔAngle) obtained from Group G1 were subtracted from the splinted group values prior to statistical analysis. All analyses were conducted by the same operator following a pre-defined standard protocol.

*Statistical Analysis*

Normality assumptions by group were assessed using the Shapiro-Wilk test, and homogeneity of variances was evaluated using Levene's test. For point-based RMS deviation and ΔAngle (°), which did not follow a normal distribution, non-parametric factorial ART analysis was applied; one-way ANOVA was used for surface RMS deviation, which followed a normal distribution. For inter-regional comparisons in the non-splinted group, the Kruskal-Wallis H test was used, and multiple comparisons were conducted using Bonferroni-corrected Z tests. Significant inter-group differences in all analyses were identified using Bonferroni-corrected multiple comparison tests; effect sizes were reported as partial eta-squared (ηp²), and results were presented using a lettering scheme. The significance level was set at *p<*0.05; all analyses were performed using R software (R Core Team, 2025; R Foundation for Statistical Computing, Vienna, Austria) with the ARTool, emmeans, and ggplot2 packages.

## Results

The G1 group represented the inherent measurement error of the SIRIOS™ scanner, and all values obtained from the splinted groups were corrected by subtracting this error. Point-based RMS deviation did not differ significantly among arch regions (H=2.034, *p=*0.362), while ΔAngle values were significantly higher in the anterior and premolar regions than in the molar region (H=53.899, *p<*0.001). Surface RMS values could only be evaluated in the molar region due to methodological constraints.

Surface RMS deviation differed significantly among the groups, with a large effect size (*F=*45.903, *p<*0.001, ηp²=0.793). Post hoc analysis demonstrated significant differences between all groups. The lowest surface deviation was observed in G5, whereas the highest was recorded in G2 as shown in Table 2 and Figure 2.

For point-based RMS deviation, a significant difference was identified among splint configurations (*F=*6.656, *p<*0.001, ηp²=0.081), while the effect of arch region was not significant (*F=*1.288, *p=*0.278). However, the splint × region interaction was significant (*F=*3.215, *p=*0.005, ηp²=0.078). Specifically, G4 exhibited significantly greater deviation than G2, G3, and G5, particularly in the anterior region as shown in Table 3.

For angular deviation, significant differences were observed among splint configurations (*F=*5.228, *p=*0.002, ηp²=0.064) and among arch regions (*F=*22.688, *p<*0.001, ηp²=0.166), while the splint × region interaction was not significant (*F=*1.756, *p=*0.109). Among the splint configurations, G2 demonstrated the highest angular deviation, whereas G5 showed the lowest. Across all groups, the anterior and premolar regions exhibited significantly greater deviation than the molar region (0.29° and 0.34° vs. 0.14°, respectively). Nevertheless, all recorded values remained below the clinically acceptable threshold of 0.4° as shown in Table 3.

In the implant-level point-based RMS analysis, splint configuration (*F=*9.605, *p<*0.001, ηp²=0.118), implant position (*F=*6.093, *p<*0.001, ηp²=0.124), and their interaction (*F=*2.901, *p<*0.001, ηp²=0.168) were all statistically significant. Implant 16 demonstrated the highest deviation, whereas implant 26 showed the lowest. At implant position 12, G4 differed significantly from all other groups.

For implant-level angular deviation, splint configuration (*F=*14.788, *p<*0.001, ηp²=0.170), implant position (*F=*20.701, *p<*0.001, ηp²=0.324), and their interaction (*F=*9.025, *p<*0.001, ηp²=0.385) were all statistically significant. Implants 24 and 12 exhibited the highest angular deviation, whereas implants 26 and 16 showed the lowest values. Notably, implant 12 in G2 demonstrated markedly greater angular deviation than the corresponding implants in the other groups as shown in Table 4.

## Discussion

The null hypothesis, which assumed that different splinting configurations would not affect the accuracy of intraoral scanning, was rejected. Splint configuration significantly influenced scanning accuracy across all measured parameters. Given that improved scanning accuracy directly contributes to the passive fit and long-term success of implant-supported prostheses, appropriate planning of splint configuration is of critical clinical importance [4,11]. While previous studies have investigated splinting approaches using various materials, scan body designs, and connection protocols, no study has examined configuration-dependent accuracy variations within a single splinting system designed for complete-arch implant workflows.

The effect of splinting on scanning accuracy was found to be parameter-dependent. While all splinted groups demonstrated superior performance in angular deviation compared to the control, improvements in surface RMS and point-based deviation varied according to configuration. This finding challenges the general assumption that splinting universally enhances scanning accuracy [5]. Importantly, splinting all connection points does not necessarily result in improved accuracy. In fact, the lowest surface RMS deviation was observed in G5, whereas the highest was recorded in G2. The improved performance of G5 may be attributed to a more balanced force distribution created by the cross-arch connection geometry, which likely reduces cumulative error accumulation. In contrast, the higher deviation observed in G2 may be associated with the broader splint design spanning both posterior implants, potentially amplifying cumulative scanning errors.

Regarding point-based deviation, higher values were observed in the G4 group. This may be explained by the radial branching geometry originating from the center, which increases moment arm length and may introduce asymmetric polymerization shrinkage stresses at the connection interfaces. The notably higher deviation in the anterior region may be related to the absence of a continuous splint line in G4, unlike the other configurations, which may have reduced the scanner’s ability to establish a stable reference framework during image stitching, resulting in error accumulation in the anterior region. At the implant level, implant 16 demonstrated the highest deviation, whereas implant 26 showed the lowest. This difference may be related to scanning path dependency. Implants located closer to the initial scanning position, such as implant 26, are likely less affected by cumulative error accumulation, whereas implants located toward the end of the scanning path, such as implant 16, may be more susceptible to error propagation [18].

For angular deviation, arch position was identified as an independent factor from splint configuration. The higher deviation observed in the anterior and premolar regions may be associated with the curvature of the anterior arch and cumulative error propagation along the scanning path. In G2, the linear geometry following the arch contour may have increased angular deviation by inducing micro-movements along the anterior-posterior direction. Conversely, the cross-arch connection in G5 may have provided bidirectional stabilization, reducing directional drift and thereby decreasing angular deviation. Notably, all values remained below the clinically accepted threshold of 0.4° reported in the literature [19].

When all three parameters are considered together, no configuration demonstrated uniform accuracy across the entire arch. However, G5 showed a more balanced performance profile, with the lowest surface RMS deviation and relatively stable angular deviation, suggesting that it may be the most favorable option in the presence of parallel implant configurations. Therefore, splint configurations should not be evaluated based on a single accuracy parameter, but rather through a comprehensive assessment of all dimensional accuracy outcomes [20].

To the best of our knowledge, this study is among the first to evaluate the effect of different splinting patterns on intraoral scanning accuracy in an All-on-6 configuration using the Straumann EXACT™ system, thereby contributing to the growing body of evidence on the influence of splint configuration in digital implant impressions. This study has several limitations. An *in vitro* design was used as clinical variables such as saliva, soft tissue mobility, patient motion, and limited mouth opening were excluded. These variables would introduce additional variability and affect the isolation of the splinting configuration effect. A single reference model was used to ensure methodological standardization. The measurement error of the Solutionix C500 reference scanner has been certified at 1 µm. Therefore, scan-to-scan variability in the reference dataset was considered negligible at the precision levels evaluated in this study. A single intraoral scanner was used to prevent device-related variability from influencing the results. The Straumann SIRIOS™ scanner was selected because all implant components used in this study belong to the same manufacturer and the EXACT™ system has been validated with this scanner by the manufacturer, which was expected to support more accurate scan body recognition within the native software environment. A parallel implant configuration was used. The inclusion of angulated implants would have introduced an additional variable and affected the isolation of the splinting effect. However, the accuracy of splinting in angulated implant configurations should be investigated in future studies. Only EXACT™ system components were used for splinting. The manufacturer recommends a standard connection configuration for six implants. However, the number, position, angulation, and inter-implant distance vary considerably among patients in clinical practice. Therefore, exact replication of this protocol is not feasible in every case. This study aimed to evaluate the effect of different connection configurations within the same system on impression accuracy, rather than comparing different splinting materials. All scanning procedures were performed by a single operator to control for inter-operator variability, as differences in experience level and scanning strategy among operators could influence outcomes. However, this limits the generalizability of the findings to operators with varying levels of experience. Future studies should evaluate different intraoral scanner systems, angulated implant configurations, multiple operators, and clinical conditions.

## Conclusions

Within the limitations of this *in vitro* study, different splinting protocols were shown to significantly influence intraoral scanning accuracy. The effect of splint design was found to be parameter-dependent, with surface-based, point-based, and angular deviation metrics demonstrating variable performance across different configurations. No single protocol demonstrated consistent superiority across all accuracy parameters. Further studies incorporating different implant distributions, intraoral scanner systems, and clinical patient conditions are required to validate these findings and enhance their clinical applicability. 

## Figures and Tables

**Table 1:** Splinting configurations used in the study groups.

**Group**	**Splinting Configuration**
G1 (Control)	Non-splinted; only BASE scanbodies were used.
G2	V-Shape LINK (12, 22) and Short LINK (16, 26).
G3	Short LINK (12, 22) and Long LINK (14, 24); without posterior connection at 16, 26.
G4	Independent segmental splinting. Right: Short LINK (12, 14), Long LINK (16). Left: Short LINK (22, 24), Long LINK (26).
G5	Short LINK (14, 22) and Long LINK (16, 24); without connection at 12, 26.

**Table 2:** Distribution of corrected surface RMS deviation values by splint group.

**Group**	**Mean ± SD (mm)**	*F*	*p*	**ηp²**
G2	1.27 ± 0.10ᵈ	45.903	<0.001	0.793
G3	0.85 ± 0.08ᵇ			
G4	1.01 ± 0.16ᶜ			
G5	0.62 ± 0.15ᵃ			

G1 values were used as the correction baseline and are therefore not included in this table. Mean±SD; F: One-way ANOVA; ηp^2^: Partial eta-squared. a-d: Groups sharing a common letter do not differ significantly (Bonferroni post-hoc test).

**Table 3:** Distribution of corrected point-based RMS deviation and ΔAngle(°) values by splint group and arch region.

	**G2**	**G3**	**G4**	**G5**	**Total**
**Point Based RMS Deviation (mm)**					
Anterior	0.02 (0.00-0.06)ᴬ	0.04 (0.00-0.11)ᴬ	0.10 (0.02-0.14)ᴮ	0.04 (0.01-0.17)ᴬ	0.04 (0.00-0.17)
Molar	0.04 (0.00-0.21)ᴬ	0.03 (0.00-0.11)ᴬ	0.05 (0.00-0.27)ᴬ	0.03 (0.01-0.14)ᴬ	0.04 (0.00-0.27)
Premolar	0.02 (0.00-0.07)ᴬ	0.04 (0.00-0.18)ᴬ	0.04 (0.00-0.15)ᴬ	0.04 (0.01-0.12)ᴬ	0.04 (0.00-0.18)
Total	0.02 (0.00-0.21)ᵃ	0.03 (0.00-0.18)ᵃ	0.05 (0.00-0.27)ᵇ	0.04 (0.01-0.17)ᵃ	0.04 (0.00-0.27)
**ΔAngle (°)**					
Anterior	0.73 (0.11-2.17)	0.28 (0.00-1.74)	0.53 (0.01-3.13)	0.19 (0.01-1.51)	0.29 (0.00-3.13)ᵇ
Molar	0.19 (0.00-0.76)	0.11 (0.01-0.81)	0.15 (0.01-0.38)	0.10 (0.00-0.28)	0.14 (0.00-0.81)ᵃ
Premolar	0.40 (0.03-1.91)	0.29 (0.01-0.87)	0.24 (0.02-1.83)	0.36 (0.07-1.37)	0.34 (0.01-1.91)ᵇ
Total	0.26 (0.00-2.17)ᶜ	0.19 (0.00-1.74)ᵃ	0.24 (0.01-3.13)ᵇ	0.16 (0.00-1.51)ᵃ	0.23 (0.00-3.13)

Median (min.-max.). A-B: Interaction groups sharing a common letter do not differ significantly (Bonferroni post-hoc test). a-c: Main effect groups sharing a common letter do not differ significantly (Bonferroni post-hoc test). Point Based RMS: Splint effect F=6.656, p<0.001, ηp^2^=0.081; Region effect F=1.288, p=0.278, ηp^2^=0.011; Interaction F=3.215, p=0.005, ηp^2^=0.078. ΔAngle(°): Splint effect F=5.228, p=0.002, ηp^2^=0.064; Region effect F=22.688, p<0.001, ηp^2^=0.166; Interaction F=1.756, p=0.109, ηp^2^=0.044.

**Table 4:** Distribution of corrected implant-based point RMS deviation and ΔAngle values by splint group and implant position.

**Implant**	**G2**	**G3**	**G4**	**G5**	**Total**
**Point Based RMS Deviation (mm)**					
12	0.01 (0.00-0.06)ᴬ	0.05 (0.01-0.11)ᴬ	0.11 (0.02-0.14)ᴮ	0.04 (0.01-0.08)ᴬ	0.05 (0.00-0.14)ᵇ
14	0.02 (0.01-0.07)ᴬ	0.04 (0.00-0.13)ᴬ	0.06 (0.01-0.15)ᴬ	0.04 (0.01-0.12)ᴬ	0.03 (0.00-0.15)ᵃ
16	0.07 (0.01-0.21)ᴬ	0.03 (0.00-0.11)ᴬ	0.10 (0.01-0.27)ᴮ	0.08 (0.03-0.14)ᴬᴮ	0.06 (0.00-0.27)ᵇ
22	0.02 (0.00-0.05)ᴬ	0.03 (0.00-0.08)ᴬ	0.08 (0.02-0.14)ᴬ	0.05 (0.01-0.17)ᴬ	0.03 (0.00-0.17)ᵃ
24	0.02 (0.00-0.05)ᴬ	0.04 (0.00-0.18)ᴬ	0.03 (0.00-0.05)ᴬ	0.05 (0.01-0.12)ᴬ	0.04 (0.00-0.18)ᵃ
26	0.03 (0.00-0.07)ᴮ	0.03 (0.01-0.06)ᴬ	0.03 (0.00-0.07)ᴬ	0.02 (0.01-0.04)ᴬ	0.02 (0.00-0.07)ᵃ
Total	0.02 (0.00-0.21)ᵃ	0.03 (0.00-0.18)ᵃ	0.05 (0.00-0.27)ᵇ	0.04 (0.01-0.17)ᵃ	0.04 (0.00-0.27)
**ΔAngle (°)**					
12	1.53 (1.10-2.17)ᴮ	0.23 (0.00-0.87)ᴬ	0.53 (0.01-3.13)ᴬ	0.13 (0.01-1.51)ᴬ	0.35 (0.00-3.13)ᶜ
14	0.20 (0.03-0.42)ᴬ	0.25 (0.01-0.87)ᴮ	0.12 (0.02-1.83)ᴬ	0.43 (0.08-1.37)ᴮ	0.19 (0.01-1.83)ᵇ
16	0.22 (0.03-0.76)ᴬ	0.11 (0.03-0.81)ᴮ	0.23 (0.01-0.38)ᴬ	0.10 (0.00-0.28)ᴮ	0.17 (0.00-0.81)ᵃ
22	0.15 (0.11-0.36)ᴬ	0.32 (0.01-1.74)ᴮ	0.51 (0.10-2.34)ᴮ	0.47 (0.07-1.05)ᴮ	0.27 (0.01-2.34)ᵇ
24	0.75 (0.36-1.91)ᴮ	0.34 (0.09-0.56)ᴬ	0.68 (0.04-1.56)ᴮ	0.30 (0.07-0.73)ᴬ	0.42 (0.04-1.91)ᶜ
26	0.12 (0.00-0.34)ᴬ	0.10 (0.01-0.27)ᴮ	0.12 (0.01-0.24)ᴬ	0.11 (0.00-0.27)ᴮ	0.11 (0.00-0.34)ᵃ
Total	0.26 (0.00-2.17)ᶜ	0.19 (0.00-1.74)ᵃ	0.24 (0.01-3.13)ᵇ	0.16 (0.00-1.51)ᵃ	0.23 (0.00-3.13)

Median (min.-max.). A-B: Interaction groups sharing a common letter do not differ significantly (Bonferroni post-hoc test). a-c: Main effect groups sharing a common letter do not differ significantly (Bonferroni post-hoc test). Point Based RMS: Splint effect F=9.605, p<0.001, ηp^2^=0.118; Implant effect F=6.093, p<0.001, ηp^2^=0.124; Interaction F=2.901, p<0.001, ηp^2^=0.168. ΔAngle(°): Splint effect F=14.788, p<0.001, ηp^2^=0.170; Implant effect F=20.701, p<0.001, ηp^2^=0.324; Interaction F=9.025, p<0.001, ηp^2^=0.385.

**Figure 1:**
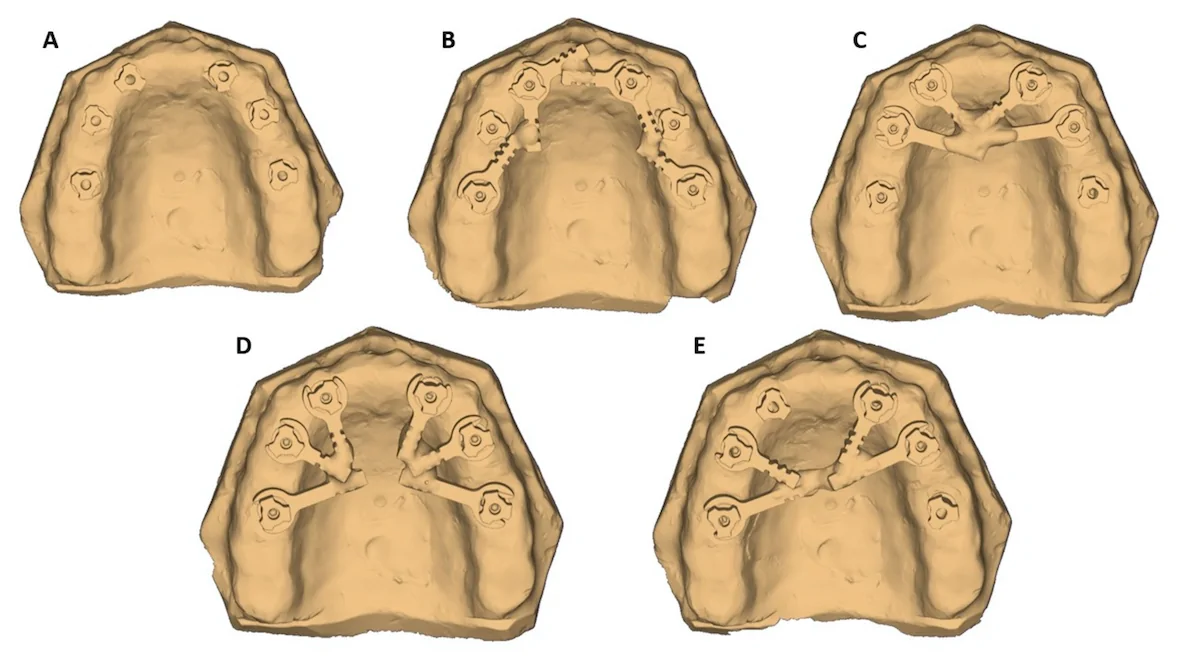
Splinting configurations used in the study groups. (A) G1: Non-splinted control; only BASE scanbodies were placed. (B) G2: Bilateral splinting with a V-Shape LINK connecting implants 12 and 22, and a Short LINK connecting implants 16 and 26. (C) G3: Anterior splinting with a Short LINK connecting implants 12 and 22, and a Long LINK connecting implants 14 and 24; implants 16 and 26 were left unsplinted. (D) G4: Independent segmental splinting; on the right side, a Short LINK connected implants 12 and 14, and a Long LINK was placed at implant 16; on the left side, a Short LINK connected implants 22 and 24, and a Long LINK was placed at implant 26. (E) G5: Cross-arch splinting with a Short LINK connecting implants 14 and 22, and a Long LINK connecting implants 16 and 24; implants 12 and 26 were left unsplinted.

**Figure 2:**
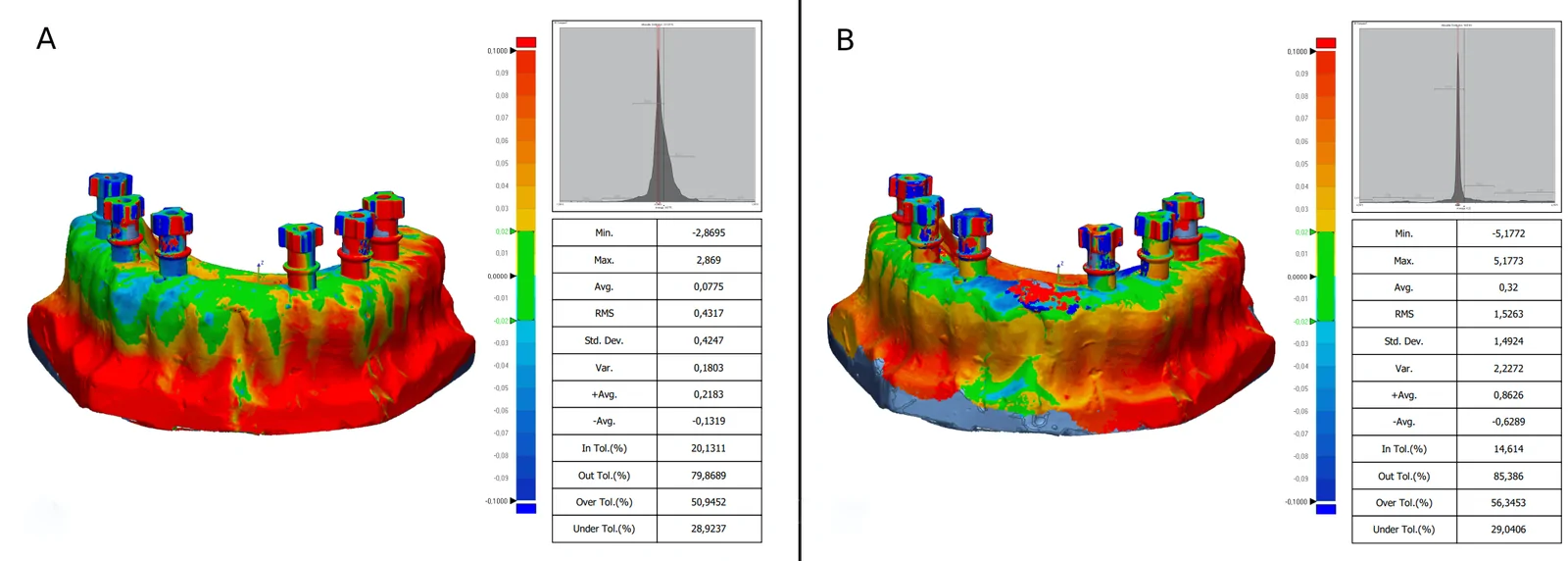
Surface RMS deviation color maps of the scan with the highest deviation within G2 (A) and the scan with the lowest deviation within G5 (B), obtained by superimposition against the reference dataset using Geomagic Control X. The color scale represents deviations ranging from -0.100mm (blue) to +0.100 mm (red). RMS: root mean square.

## Data Availability

The datasets generated and analyzed during the current study are available from the corresponding author upon reasonable request.
